# Cefiderocol to manage chronic, multi-drug-resistant *Burkholderia cepacia* complex infection in a patient with cystic fibrosis: a case report

**DOI:** 10.1099/acmi.0.000413

**Published:** 2022-10-07

**Authors:** Clemency Nye, Jamie Duckers, Rishi Dhillon

**Affiliations:** ^1^​ Public Health Wales Microbiology Cardiff, University Hospital of Wales, Heath Park, Cardiff CF14 4XW, UK; ^2^​ All Wales Adult Cystic Fibrosis Centre, University Hospital Llandough, Cardiff CF64 2XX, UK

**Keywords:** *Burkholderia cenocepacia*, cefiderocol, cystic fibrosis, multi-drug resistance

## Abstract

In cystic fibrosis (CF) patients, Gram-negative *Burkholderia cepacia* complex (Bcc) infections are associated with recurrent pulmonary exacerbations. Bcc organisms are innately resistant to many antibiotics, and infection with *B. cenocepacia* is a contraindication to lung transplantation. We report a CF patient with severe lung disease, colonized with Bcc, with a history of around nine exacerbations per year for over 10 years, for whom antibiotic regimens (including targeted and broad-spectrum antibiotics) had not cleared infection or extended the interval between exacerbations. After receiving a 2 week cefiderocol-containing regimen, the patient remained stable for more than 5 months without the need for additional antibiotics or hospital admissions for respiratory exacerbations.

## Introduction

Patients with cystic fibrosis (CF) are highly susceptible to chronic pulmonary infections, and persistent lower airway infection is the major cause of progressive lung damage, morbidity and mortality. CF patients often require repeated and intensive antibiotic therapy to maintain lung function and reduce exacerbations [1].

In this patient population, pulmonary infections dominated by *Burkholderia cepacia* complex (Bcc) organisms, a group of at least 22 known species of Gram-negative bacteria, are particularly difficult to treat [1, 2]. These organisms live within biofilms and are innately resistant to a range of antibiotics and able to acquire resistance against many more [1, 3, 4].

Bcc typically establishes chronic infection in patients with CF [1, 5]. Bcc is less common than the other major Gram-negative pathogen in CF, *Pseudomonas aeruginosa *, with a prevalence of 2.6 % in the UK in 2019 [6]. However, compared with chronic *Pseudomonas* infection, lung damage is more rapid and life expectancy is more significantly reduced [7]. As well as chronic infection, Bcc can occasionally cause an acute necrotizing pneumonia with respiratory failure and sepsis, which is termed ‘cepacia syndrome’ [7]. Cepacia syndrome has an extremely poor prognosis, being fatal in the majority of cases. It can occur at any point after the initial infection, including years later [7].

The clinical course of Bcc infection is often unpredictable and may be related to the pathogenicity of the dominant Bcc strain [8]. The most common species in CF patients are *Burkholderia cenocepacia* and *B. multivorans *, accounting for 85–97 % of Bcc infections [9]. *B. cenocepacia* is more likely than *B. multivorans* to establish chronic infection [10], and is associated with worse respiratory outcomes and poorer survival rates [8, 9]. Critically, *B. cenocepacia* colonization is a contraindication to lung transplantation, thus denying patients with CF a potentially life-saving procedure [1, 11].

Treatment of Bcc is challenging, with inherent antibiotic resistance and susceptibility testing issues, and there have been no randomized controlled trials of treatments for respiratory exacerbations in patients with CF chronically infected with Bcc [1].

CF patients colonized with Bcc pathogens rely heavily upon antimicrobial therapy to tackle and control recurrent infections, and there is a recognized need to optimize antibiotic strategies to manage CF pulmonary exacerbations due to Gram-negative bacilli such as Bcc [1, 11].

Cefiderocol is a novel antibiotic – a siderophore–cephalosporin conjugate, active against a variety of multidrug-resistant (MDR) bacteria encountered by CF patients [12]. Cefiderocol is indicated for Gram-negative infections in adults with limited treatment options [13]. The CREDIBLE-CR study was a small phase-3, open-label, pathogen-focused study in adults with life-threatening carbapenem-resistant (CR) Gram-negative infections [14]. This study provided descriptive evidence of the efficacy and safety of cefiderocol in CR Gram-negative infections in a heterogeneous patient population. The clinical and microbiological outcomes were similar between cefiderocol and best available therapy, except for metallo-beta-lactamase-producing organisms where cefiderocol was substantially better.

To date, available clinical data on cefiderocol in patients with CF and Gram-negative infections are based on compassionate-use cases [15]. Worldwide (data for November 2017 to December 2019), 27 patients with CF have been treated via the cefiderocol compassionate-use-programme, of whom 70 % showed a clinical response to treatment. Nine of the 27 patients had *Burkholderia* pathogens.

We are not aware of any published case reports on the use of cefiderocol in the treatment of Bcc infection in patients with CF. Here we report a clinical case of a CF patient with chronic Bcc colonization and recurrent pulmonary exacerbations, managed with cefiderocol.

## Case report

Our patient is a 39-year-old man with CF with chronic Bcc colonization and a history of recurrent pulmonary exacerbations linked to *B. cenocepacia* infection. The patient has pancreatic insufficiency, CF-diabetes and diabetes-associated renal insufficiency, and is on insulin therapy. He has a portacath (implantable central venous access device) and a percutaneous endoscopic gastrostomy (PEG) tube to support chronic CF disease management. In addition to persistent Bcc, the patient is colonized with *Staphylococcus aureus* that is sensitive to flucoxacillin. *S. aureus* has been isolated persistently since 2016 and the patient has been colonized with *B. cenocepacia* since at least 2007, with the ET-12 strain – a highly transmissible, epidemic strain – isolated recurrently in all sputum samples. The patient is *P. aeruginosa *-negative and the patient records identify no other significant positive microbiology.

This patient is not a candidate for lung transplantation due to his *B. cenocepacia* infection history and he is supported by our palliative care team for ongoing joint and chest pain. His pain management involves controlled morphine therapy.

The patient’s CF-related treatments include Kaftrio (ivacaftor, tezacaftor and elexacaftor), which he has been receiving long-term, and long-term flucloxacillin. Although not maintained on any nebulized antibiotics due to his inability to tolerate such therapies, this patient has received repeated and recurrent courses of intravenous (IV) antibiotics targeting *B. cenocepacia *, which have included ceftazidime, meropenem, co-trimoxazole, temocillin, ceftazime-avibactam and ceftolazone-tazobactam.

Historically, this patient has required around five courses of IV antibiotics a year (around 70 days a year on IV antibiotic therapies), in addition to around four courses of oral antibiotics per year, to manage his pulmonary exacerbations. These treatment regimens have not succeeded in improving the patient’s baseline symptoms or extending the interval between his exacerbations.

**Fig. 1.**
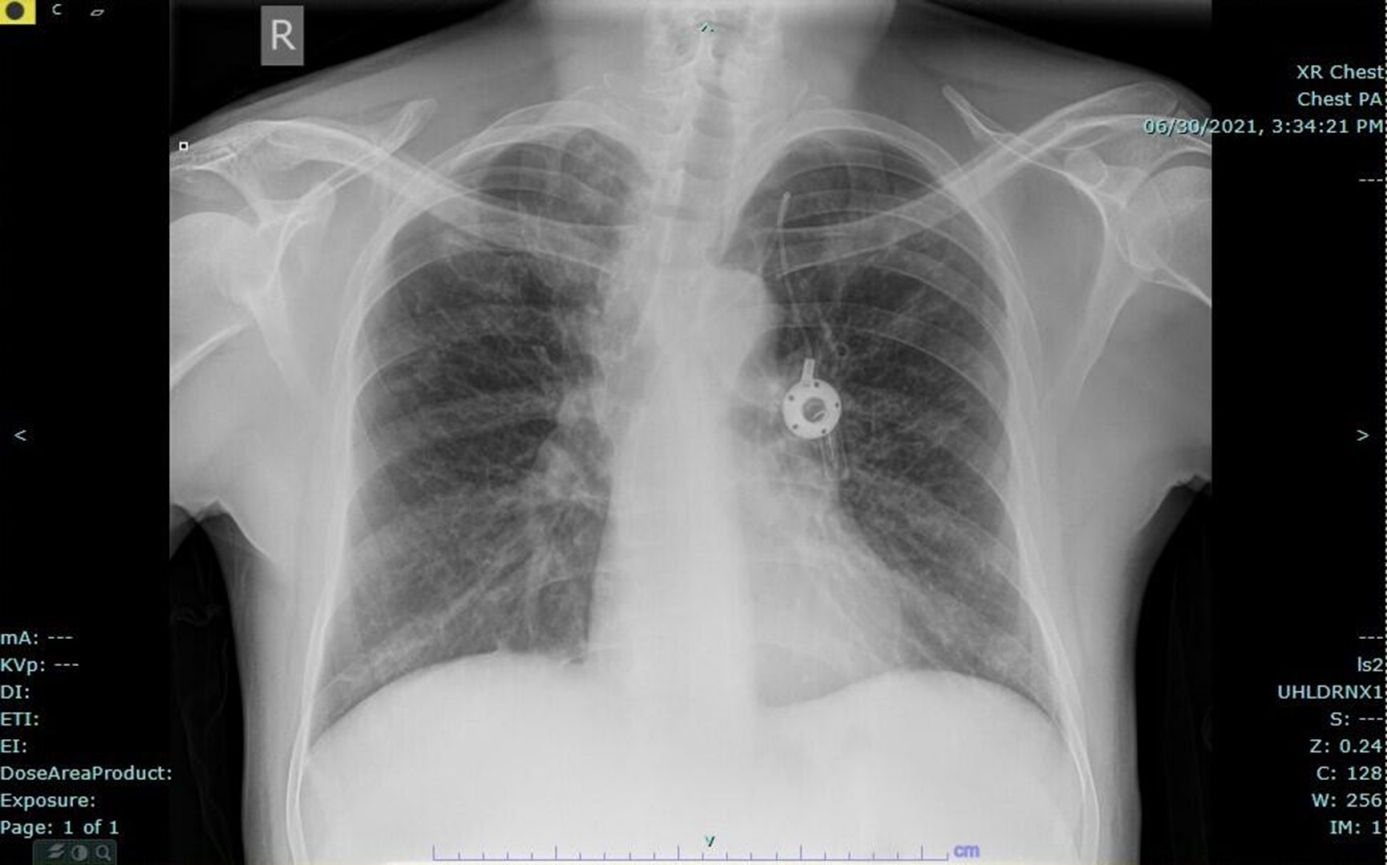
Chest x-ray of our patient at admission with respiratory exacerbation in July 2021.

In July 2021 the patient was admitted to hospital with a new pulmonary exacerbation. On this occasion, the evidence suggesting an infective exacerbation was that the patient had increased respiratory symptoms, a rise in C-reactive protein (CRP) and new patchy airspace shadowing on chest X-ray (see Fig. 1 for chest X-ray on admission). His baseline FEV1 was 0.49 litres (12 % predicted), his baseline CRP was 10 mg l^−1^ (falling to 4 mg l^−1^ during the exacerbation) and his white cell count was 6.3×10^9^ l^−1^.

Fungal markers were normal and the patient’s sputum identified *B. cenocepacia* and light growth of *S. aureus* sensitive to flucloxacillin.

Given the patient’s history of Bcc infection and microbiology at admission, cefiderocol susceptibility testing was undertaken, showing that the organism was susceptible to cefiderocol *in vitro* with an MIC of 0.032 mg l^−1^. In light of previous antibiotic exposure and lack of efficacy of most recent regimens, the patient was therefore managed with IV cefiderocol and tobramycin. This treatment regimen was administered during a 2 week hospital admission, and comprised cefiderocol at 1.5 g TDS and tobramycin 80 mg day. Reduced doses were given due to the patient’s renal insufficiency. In addition to the 2 week course of cefiderocol–tobramcycin treatment, the patient’s long-term oral flucloxacillin therapy was adjusted from 1 g BD to 1 g QDS PO. There were no adverse events experienced with the use of cefiderocol.

During treatment, the patient’s FEV1 remained unchanged from baseline, and did not deteriorate further following completion of the course of treatment. There was no change in the patient’s morphine requirements during and after the treatment course. However, while no formal pain score assessments were made, during and following the 2 week treatment course the patient noted, and actively reported, improvements in pain. Subsequent to his treatment course, the patient considers that, symptomatically, his response to this treatment regimen has been better than that to any previous antibiotic regimens he has received for exacerbations either as an inpatient or as an outpatient.

Of particular note, since receiving this cefiderocol-based treatment in July 2021, the patient has not required further courses of antibiotics or hospital admissions due to pulmonary exacerbations. As of December 2021, the patient continues to be managed at home.

## Discussion

The case presented here is all too familiar of CF – a condition typified by chronic, recurrent MDR infection and pulmonary exacerbations that contribute to progressive lung damage and to CF morbidity and mortality [1].

As noted, this patient is not a candidate for lung transplantation due to his chronic *B. cenocepacia* colonization, and his CF management is palliative – based on a goal of optimizing quality of life in the face of a progressive condition which for him is without a definitive treatment option.

Previous Bcc-associated pulmonary exacerbations contributed to this patient being hospitalized on a regular basis, with admissions almost monthly in the year prior to the excacerbation reported here. For this patient, persistent and recurrent MDR Gram-negative infection has meant that for around 2 months of every year, he has needed to receive IV antibiotics that have had little impact on his chronic Bcc colonization or on his pattern of pulmonary exacerbations and hospital admissions.

Treatment with a cefiderocol-based regimen in July 2021 appears to have helped stabilize the patient in terms of pulmonary exacerbations. Importantly, the demonstration of a much extended interval between such acute episodes – as evidenced by no admission for IV antibiotics and no pulmonary exacerbations from July to December (a period of 5 months without admission at the time of writing) is, for this patient, a remarkable change.

Limitations of this case study include the fact that sputum samples are not available for the period following the patient’s discharge, although we would not expect to achieve clearance of Bcc, given the chronic and persistent nature of Bcc colonization once established in CF patients [6]. A further limitation is that no formal pain-score assessments were made to objectively determine the impact of the cefiderocol-based treatment regimen on the patient’s CF-associated pain.

However, for a CF patient receiving palliative care, with a history of multiple annual hospital admissions for intensive IV antibiotic therapies that do not appear to have controlled chronic Bcc colonization, the impact of cefiderocol therapy in supporting some remission from exacerbations and reducing this patient’s hospitalizations and exposure to repeated courses of antibiotic therapies is noteworthy. The patient himself reported that this treatment appeared to offer better symptomatic control – and although subjective, such observations point to an improved quality of life for this patient, who has a limited life expectancy.

The management of pulmonary infections, and Bcc infection specifically, requires that clinicians assess each person individually, taking into account symptomatic changes*,* previous clinical responses and their own experiences [3]. Evidence from the literature, which to date is largely founded on *in vitro* studies or case reports, supports that cefiderocol is an antibiotic that appears to expand the treatment options for CF patients colonized with MDR Gram-negative bacilli [15–20].

To conclude, in an area in which there is a paucity of robust data regarding how best to optimize management of MDR Gram-negative infection in CF patients, and in which there is a high unmet need to address chronic Bcc colonization in CF patients – particularly given the ramifications of Bcc colonization on curative transplant options [1, 5, 6, 11] – our case report contributes new insights on managing MDR Bcc and adds to the understanding of where agents such as cefiderocol may be utilized to support improved outcomes for patients with CF.

## References

[1] Lord R, Jones AM, Horsley A (2020). Antibiotic treatment for *Burkholderia cepacia* complex in people with cystic fibrosis experiencing a pulmonary exacerbation. Cochrane Database Syst Rev.

[2] Jin Y, Zhou J, Zhou J, Hu M, Zhang Q (2020). Genome-based classification of *Burkholderia cepacia* complex provides new insight into its taxonomic status. Biol Direct.

[3] Horsley A, Jones AM, Lord R (2016). Antibiotic treatment for *Burkholderia cepacia* complex in people with cystic fibrosis experiencing a pulmonary exacerbation. Cochrane Database Syst Rev.

[4] Horsley A, Webb K, Bright-Thomas R, Govan J, Jones A (2011). Can early *Burkholderia cepacia* complex infection in cystic fibrosis be eradicated with antibiotic therapy?. Front Cell Infect Microbiol.

[5] Regan KH, Bhatt J (2019). Eradication therapy for *Burkholderia cepacia* complex in people with cystic fibrosis. Cochrane Database Syst Rev.

[6] Trust CF (2019). UK cystic fibrosis registry annual data report 2019. https://www.cysticfibrosis.org.uk/the-work-we-do.

[7] Jones AM, Dodd ME, Govan JRW, Barcus V, Doherty CJ (2004). *Burkholderia cenocepacia* and *Burkholderia multivorans*: influence on survival in cystic fibrosis. Thorax.

[8] France MW, Dodd ME, Govan JR, Doherty CJ, Webb AK (2008). The changing epidemiology of *Burkholderia* species infection at an adult cystic fibrosis centre. J Cyst Fibros.

[9] Drevinek P, Mahenthiralingam E (2010). *Burkholderia cenocepacia* in cystic fibrosis: epidemiology and molecular mechanisms of virulence. Clin Microbiol Infect.

[10] Zlosnik JEA, Zhou G, Brant R, Henry DA, Hird TJ (2015). *Burkholderia* species infections in patients with cystic fibrosis in British Columbia, Canada. 30 years’ experience. Ann Am Thorac Soc.

[11] Belcher R, Zobell JT (2021). Optimization of antibiotics for cystic fibrosis pulmonary exacerbations due to highly resistant nonlactose fermenting Gram negative bacilli: meropenem-vaborbactam and cefiderocol. Pediatr Pulmonol.

[12] Gavioli EM, Guardado N, Haniff F, Deiab N, Vider E (2021). Does cefiderocol have a potential role in cystic fibrosis pulmonary exacerbation management?. Microb Drug Resist.

[13] Cefiderocol. Cefidericol summary of product characteristics. https://www.medicines.org.uk/emc/product/11771#gref.

[14] Bassetti M, Echols R, Matsunaga Y, Ariyasu M, Doi Y (2021). Efficacy and safety of cefiderocol or best available therapy for the treatment of serious infections caused by carbapenem-resistant Gram-negative bacteria (CREDIBLE-CR): a randomised, open-label, multicentre, pathogen-focused, descriptive, phase 3 trial. Lancet Infect Dis.

[15] Shionogi (2022). Shionogi data on file (cefiderocol compassionate use programme-unpublished).

[16] Warner NC, Bartelt L, Lachiewicz A, Lachiewicz A, Tompkins KM (2019). 730. Cefiderocol for the treatment of *Achromobacter xylosoxidans* infections in two lung transplant patients with cystic fibrosis. Open Forum Infect Dis.

[17] Warner NC, Bartelt LA, Lachiewicz AM (2020). Cefiderocol for the treatment of adult and pediatric patients with cystic fibrosis and *Achromobacter xylosoxidans* infections. Clin Infect Dis.

[18] Gainey AB, Burch A-K, Brownstein MJ, Brown DE, Fackler J (2020). Combining bacteriophages with cefiderocol and meropenem/vaborbactam to treat a pan-drug resistant *Achromobacter* species infection in a pediatric cystic fibrosis patient. Pediatr Pulmonol.

[19] Firoz A, Haris M, Hussain K, Raza M, Verma D (2021). Can targeting iron help in combating chronic *Pseudomonas* infection? A systematic review. Cureus.

[20] Burnard D, Robertson G, Henderson A, Falconer C, Bauer MJ (2021). *Burkholderia pseudomallei* clinical isolates are highly susceptible *in vitro* to cefiderocol, a siderophore cephalosporin. Antimicrob Agents Chemother.

